# Rapidly Progressive Fatal Pneumococcal Meningitis in a Fully Immunized Child With a History of Facial Bone Fractures

**DOI:** 10.7759/cureus.59204

**Published:** 2024-04-28

**Authors:** Brianna N Stanley, Haya B Rizvi, Hanna S Sahhar

**Affiliations:** 1 Pediatrics, Edward Via College of Osteopathic Medicine, Spartanburg, USA; 2 Pediatrics, Edward Via College of Osteopathic Medicine, Blacksburg, USA; 3 Pediatric Intensive Care Unit, Spartanburg Regional Healthcare System, Spartanburg, USA

**Keywords:** bacterial meningitis, brain herniation, septic thrombophlebitis, post-traumatic meningitis, pneumococcal meningitis

## Abstract

Meningitis is the inflammation of meninges either septic or aseptic depending on the source of infection. Typical signs and symptoms of meningitis in children include fever, headache, neck stiffness, nuchal rigidity represented by positive Kernig and Brudzinski signs, photophobia, nausea, vomiting, confusion, lethargy, and irritability. Bacterial meningitis is commonly caused by *Streptococcus pneumoniae* in children over the age of three months. Although there has been a decline in infections due to the introduction of the pneumococcal conjugate and pneumococcal polysaccharide vaccines, there are still reported cases of invasive pneumococcal infections mostly with non-vaccine serotypes. We report a fully immunized six-year-old male patient with a presentation of classic meningitis signs and symptoms who developed rapid progression of disease including sudden and dramatic change in physical exam and subsequent respiratory depression within 12 hours of admission. Our patient had a history of extensive traumatic facial bone fractures six months prior. Our case demonstrates a unique presentation of rapidly progressing pneumococcal meningitis due to a suspected complication of septic thrombophlebitis and subsequent brain herniation in a fully immunized patient six months after a severe traumatic facial injury.

## Introduction

Bacterial meningitis is most often caused by three bacterial organisms, *Neisseria meningitidis*, *Haemophilus influenzae*, and *Streptococcus pneumoniae*, with the last organism being the most common [1]. *S. pneumoniae *can lead to invasive meningitis in those older than three months of age and those with a history of skull trauma [1]. Infection and spread of the disease occur through respiratory droplets that can travel through the nasopharynx and lungs [2]. Eventually, with persistent infection, there is penetration into the cerebrospinal fluid (CSF) where the bacteria can quickly multiply [1,2]. Although the introduction of pneumococcal vaccinations showed a reduction in invasive disease with vaccine serotypes, there has not been an eradication in invasive disease caused by vaccine serotypes, most commonly caused by serotypes 3 and 19A [3]. Compared to other bacterial origins, pneumococcal meningitis has shown an increased mortality rate of roughly 8% in developed regions [2].

The classic meningeal signs of headache, fever, irritability, and neck pain are seen in adults and children [2]. Patients can present with a variety of other symptoms depending on their age and other comorbidities such as nausea, vomiting, altered mental status, and seizure-like activity [2]. Infants and neonates can have atypical presentations where the clinical manifestation may only show fever and irritability [2]. Physical examinations can demonstrate neck stiffness, photophobia, Kernig’s sign, Brudzinski’s sign, and a bulging fontanelle [2]. Diagnostic evaluation should be done promptly with stabilization if needed and initiation of antibiotic therapy [2]. Lumbar punctures should be performed to determine etiology in the CSF and should only be delayed if the patient is unstable [2].

Complications following bacterial meningitis are mostly neurologic, including cerebral infarction, hemorrhage, abscess, and cerebral edema [2], and more uncommonly, cerebral venous thrombosis [4] where rapid progression to brain death can occur [2,4]. Clinical manifestations of these neurological sequelae include hearing loss, visual impairments, seizures, hydrocephalus [4,5], cognitive deficits [5], and increased intracranial pressure (ICP) [5]. Those with a history of trauma, immunosuppression, and other comorbidities are at a higher risk of developing complications, specifically the association of head injury and increased ICP [2,5]. Increased ICP is associated with pupillary abnormalities, especially in those with a history of brain or facial injury [6]. The asymmetric dilation, also known as anisocoria, and decreased pupillary light reflex are associated with herniation and brainstem compression [6]. The Neurological Pupil index (NPi) is a measuring tool that assesses pupillary light reflex and is scored between 0 and 5 [6,7]. Scores greater than 3 are considered within the normal range while values less than 3 or equal to 0 are considered abnormal or non-reactive, respectively, and can be correlated with increased ICP [6]. Raised ICP can be evaluated with physical examination findings of altered mental status, a fixed and dilated pupil, and papilledema on fundoscopic examination [8]. A lumbar puncture with an opening pressure of 20 mmHg and CT imaging or magnetic resonance imaging (MRI) may show enlarged ventricles, herniation, mass effect, and effacement of cisterns and sulci [8]. 

Blunt trauma to the head such as those seen with motor vehicle accidents or sports injuries can result in skull fractures that may lead to dural tears and eventual CSF leakage due to proximity to the meninges [9]. Post-traumatic meningitis generally occurs from closed head injuries where central nervous system invasion occurs via direct invasion of bacteria from the brain to the meninges [9]. Other pathophysiology suggests that even without bone fractures the spread of infection can occur through the diploic vein [9]. *S. pneumoniae* and other mostly gram-positive bacteria have been reported to be associated with post-traumatic meningitis [9].

This article was previously presented as an abstract and poster at the 2024 Carolinas Via Research Recognition Day on February 9, 2024.

## Case presentation

**Figure 1:**
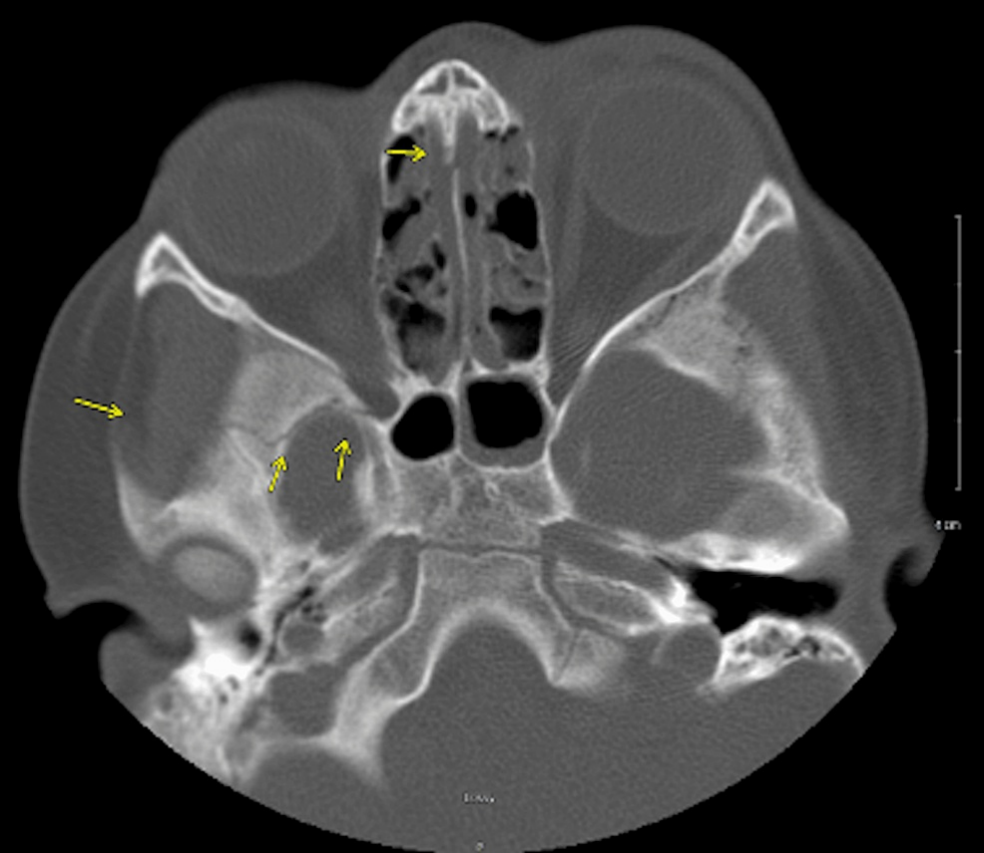
CT scan of the head CT scan of the head from six months prior when the patient was injured causing facial fractures. The following findings from the CT scan: left nasal bone fracture, bilateral impacted medial orbital wall fractures with one posteriorly on the right with the most prominent fracture, minimally displaced fracture of the right inferior temporal fossa, minimally displaced sphenoid bone, fracture of anterior cranial fossa at the cribriform plate, and oblique fracture of the left supraorbital frontal bone.

A six-year-old male patient with a history of asthma and facial trauma six months prior presented to the emergency department (ED) via emergency medical services (EMS) due to seizure-like activity at 1142. His mother reported seeing tonic/clonic-type movements during three separate episodes. She reported recent headaches since his head injury where he presented with multiple facial fractures (Figure 1). His previous head injuries did not require surgical intervention, but he did receive ceftriaxone. At the time of his injury six months prior, there was no evidence of CSF drainage. He also presented with fever, nausea, and vomiting for the past day. The patient was noted to be up to date on all his vaccinations and did not have any known drug allergies. He was febrile with a temperature of 102^o^F while in the ED, but otherwise, his vitals were unremarkable with a blood pressure of 123/82 mmHg and a heart rate of 86 beats per minute.

**Figure 2:**
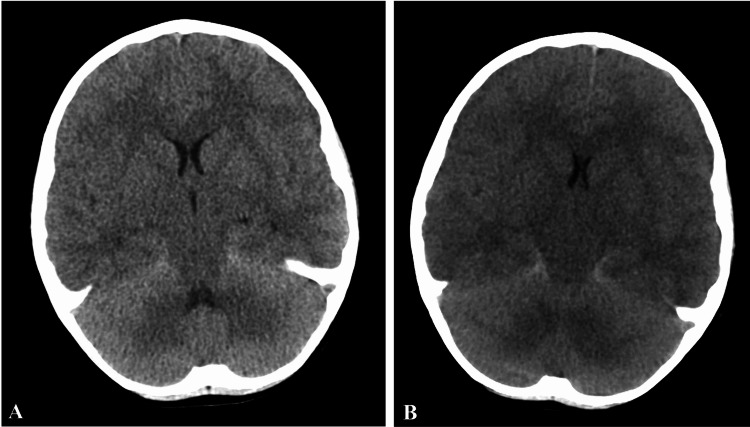
(A) Initial CT scan of the head. (B) Repeat CT scan of the head (A) Initial CT scan of the head demonstrating no acute intracranial hemorrhage, midline shift, or mass effect. (B) Repeat CT scan showing evidence of increased intracranial pressure, without evidence of acute territorial infarction or acute intracranial hemorrhage.

**Figure 3:**
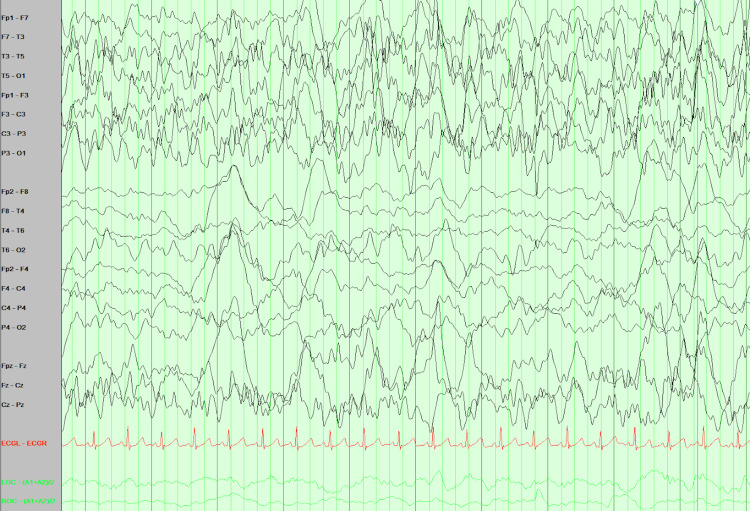
EEG findings on admission to the hospital Asymmetrical poorly organized background with relatively lower amplitudes in the right hemisphere, showing mainly high amplitudes slow delta with less abundant theta waves. No epileptiform activities in the right hemisphere. Seizures focus clearly from the left hemisphere despite muscles and other artifacts that show rhythmical 1-2 hz delta of high-amplitude spikes and sharp waves.

**Figure 4:**
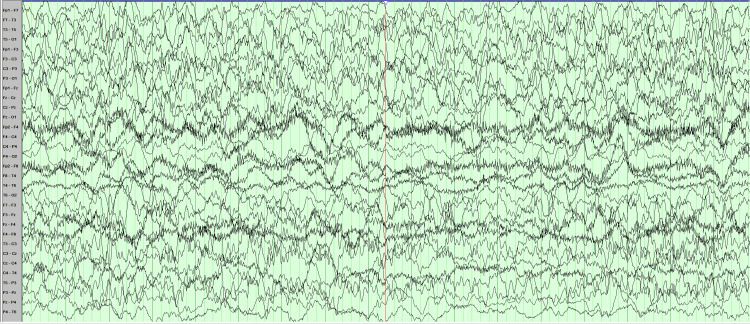
Clinical status epilepticus after hospitalization Epileptiform activities evolving to secondary generalization (focal to bilateral tonic-clonic seizure).

**Figure 5:**
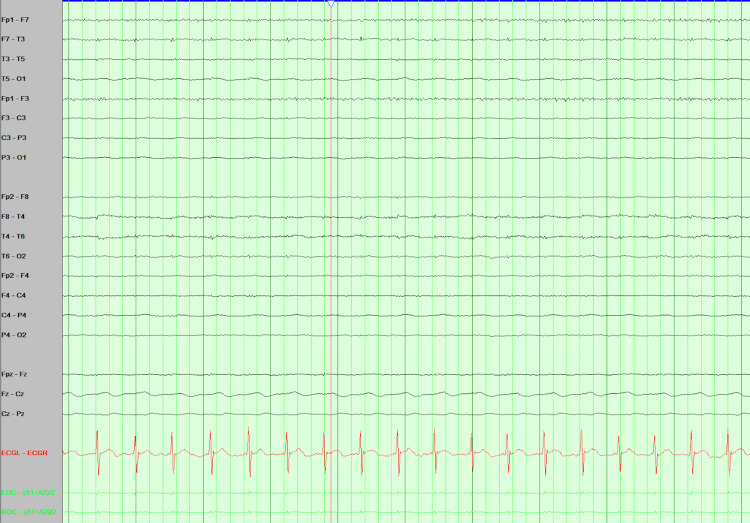
EEG ten hours after admission Diffuse background activities suppression pattern and no electrographic seizures or other epileptiform activities consistent with electro-cerebral silence.

His initial physical examination was notable for opening his eyes intermittently to his voice, moving all extremities, and being post-ictal. Laboratory investigations were significant for metabolic acidosis and an increased C-reactive protein (CRP) 14.5 mg/dL (0.0 - 0.6 mg/dL). The computed tomography (CT) scan of the head with and without contrast showed no evidence of the acute intracranial process (Figure 2A). The patient was admitted to the pediatric intensive care unit (PICU) where a lumbar puncture was completed and showed heavy growth of *S. pneumoniae* that was susceptible to ceftriaxone, levofloxacin, penicillin, and vancomycin. The *S. pneumoniae *bacteria were later identified with polymerase chain reaction (PCR) as serotype 10A. A repeat physical examination by the intensivist was significant for dry mucous membranes, delayed capillary refill, pale skin, positive neck stiffness, and positive Kernig and Brudzinski signs. His Glasgow Coma Scale (GCS) was <12 and his left pupil was larger than the right which indicates increased intracranial pressure. The patient was sluggish and tachypneic. Additional labs showed an elevated white blood cell (WBC) count 29.2 x 103/µL (5.0 - 14.5 x 103/ µL) and a decreased bicarbonate (HCO_3_) 17.7 mmol/L (23.0 -32.6 mmol/L). An electroencephalogram (EEG) was obtained pre- and post-witnessed seizure and demonstrated status epilepticus (Figures 3-5). The patient was given fosphenytoin and levetiracetam to manage his seizures.

There was a sudden change in his physical examination early the next morning at 0050, previously normal at 0031, nine hours after admission to the PICU, where his right pupil became fully dilated and non-reactive. The patient then entered respiratory depression and became severely agitated, with an oxygen saturation (SpO2) of 80% on room air. After intubation and stabilization, he was reevaluated, and both pupils were fully dilated and fixed. He was given mannitol and was found to have a decreasing NPi score (Table 1). A second CT without contrast was obtained and showed an increased intracranial pressure without evidence of infarction or hemorrhage and paranasal sinus disease (Figure 2B). Upon returning, he experienced an episode of pulseless ventricular tachycardia that required cardiopulmonary resuscitation, lidocaine, and an increased epinephrine drip to return to sinus rhythm.

**Table 1:** NPi test Neurological Pupil index (NPi) test scores can range from 0 to 5. A normal NPi score is 3.0 or greater, a score below 3.0 is considered abnormal, and a score of 0.0 is considered non-reactive. NPi at 0031, with normal NPi scores and the size of the right pupil was slightly larger than the left pupil. NPi test at 0050, after the patient had a sudden change in presentation at 0048 with dilated right pupil and respiratory depression showing an abnormal NPi score and dilated right pupil, and no change of left pupil. NPi test at 0134 shows abnormal NPi scores (non-reactive pupils) and dilated both pupils.

Time	Right NPi	Left NPi
0031	4.1	4.5
0050	0.7	3.5
0134	0.0	0.0

Roughly one hour later, the patient was noted to have a persistent GCS <3. There was noted to be progressive cerebral edema consistent with herniation along with suspected brain matter that was present in bilateral nostrils. The patient was unfortunately determined to be brain dead two days after admission following two brain death examinations.

## Discussion

The epidemiology of pneumococcal meningitis has changed since the introduction of pneumococcal vaccinations [5,10]. There is a decreased incidence of the infection of vaccine serotypes; however, the prevalence of non-vaccine serotypes has increased [10]. For instance, one case discussed the reduction in invasive pneumococcal meningitis due to the PCV 7 serotype after the introduction of the vaccine but reported increased incidents of other reported serotypes with significant morbidity and mortality [10]. Therefore, it is important to note that pneumococcal meningitis in a vaccinated individual can still result in significant diseases and consequences, as seen in our case.

Post-traumatic meningitis has been reported in a few cases following a head injury where the bacteria possibly traveled to the brain through facial sinuses [11]. These cases demonstrated head injuries of adult male patients who developed and presented with meningeal signs, post-acute [11], and chronic [9] head trauma. Although there was no statistical significance, the literature stressed the importance of pneumococcal vaccine prophylaxis in previously unvaccinated patients in those with facial fractures [11]. One of the preventative measures for patients with facial trauma includes vaccination immediately after the trauma if previously unvaccinated. In addition, antibiotics, most commonly ceftriaxone, should be given as prophylaxis if there is evidence of CSF leakage to reduce meningitis risk. Our case, although similar in terms of trauma history, is significant because of the vaccination history, young age, and rapid progression and spread of infection. The suspected brain matter that was observed in his bilateral nostrils supports our theory that facial fractures played a significant role in the spread and extent of infection in our patient. Therefore, it is important to maintain a high index of suspicion for meningitis with a similar history of facial trauma despite vaccination and antibiotics.

The uncommon complication of cerebral venous thrombosis can show similar clinical manifestations to uncomplicated meningitis and thus it can often be a missed or delayed subsequent diagnosis [4,12]. This complication occurs in 1% of patients with bacterial meningitis and is seen in those with focal neurologic deficits, coma, and ear-nose-throat infections [4]. Although the percentage is relatively low, there are life-threatening consequences in the pediatric population [12]. One study demonstrated *S. pneumoniae* to be the most common pathogen associated with cerebral venous thrombosis where 65% of patients showed growth of this organism [4]. Causes of cerebral thrombosis include hypercoagulable states, blood flow disturbances, and infection [12]. The most frequent causes were shown to be malignancy, head trauma, and hypercoagulability while infections were shown to occur in roughly 6 to 12% of cases [12]. Treatment for such thrombosis is supportive care and anticoagulation [12]. Although there is little evidence, unfractionated heparin or low-molecular-weight heparin has been utilized as anticoagulant therapy for acute cerebral venous thrombosis [12]. Our patient’s typical meningeal signs and symptoms along with his history of head injury should suggest an increased risk and predisposition to developing cerebral venous thrombosis and rapid progression to brain death. As such, further evaluation for thrombosis should be encouraged in children with such history and presentation.

## Conclusions

The six-year-old patient that was described in this case demonstrated typical meningeal signs and symptoms following a motor vehicle accident six months prior and was ultimately diagnosed with pneumococcal meningitis. This was complicated by brain herniation with the suggested theory that the patient’s facial trauma was key in the spread of infection. While pneumococcal meningitis has declined over the years, there continues to be infection primarily with non-vaccine serotypes that can lead to severe neurological complications. Therefore, it is important to recognize the clinical manifestations of meningitis in fully vaccinated patients and the consequences of facial trauma that may play a role in the rapid progression of the disease. 
